# Finite Element Analysis of a Novel Fusion Strategy in Minimally Invasive Transforaminal Lumbar Interbody Fusion

**DOI:** 10.1155/2022/4266564

**Published:** 2022-05-11

**Authors:** Zhenchuan Han, Bowen Ren, Long Zhang, Chao Ma, Jianheng Liu, Jiantao Li, Xiao Liu, Qingzu Liu, Keya Mao, Peifu Tang

**Affiliations:** ^1^Chinese PLA Medical School, Beijing 100089, China; ^2^Senior Department of Orthopedics, The Fourth Medical Centre of PLA General Hospital, Beijing 100089, China; ^3^Department of Orthopedics, PLA Rocket Force Characteristic Medical Center, Beijing 100088, China; ^4^Kunming Medical University, Kunming, Yunnan 650000, China; ^5^Key Laboratory of Modern Measurement and Control Technology, Ministry of Education, Beijing Information Science and Technology University, Beijing 100192, China

## Abstract

**Purpose:**

To evaluate the biomechanics of a novel fusion strategy (hybrid internal fixation+horizontal cage position) in minimally invasive transforaminal lumbar interbody fusion (MIS-TLIF).

**Methods:**

MIS-TLIF finite element models for three fusion strategies were created based on computed tomography images, namely, Model-A, hybrid internal fixation (ipsilateral pedicle screw and contralateral translaminar facet screw fixation)+horizontal cage position; Model-B, bilateral pedicle screw (BPS) fixation+horizontal cage position; and Model-C, BPS fixation+oblique 45° cage position. A preload of 500 N and a moment of 10 Nm were applied to the models to simulate lumbar motion, and the models' range of motion (ROM), peak stress of the internal fixation system, and cage were assessed.

**Results:**

The ROM for Models A, B, and C were not different (*P* > 0.05) but were significantly lower than the ROM of Model-INT (*P* < 0.0001). Although there were subtle differences in the ROM ratio for Models A, B, and C, the trend was similar. The peak stress of the internal fixation system was significantly higher in Model-A than that of Models B and C, but only the difference between Models A and B was significant (*P* < 0.05). The peak stress of the cage in Model-A was significantly lower than that of Models B and C (*P* < 0.01).

**Conclusion:**

Hybrid internal fixation with horizontal single cage implantation can provide the same biomechanical stability as traditional fixation while reducing peak stress on the cage and vertebral endplate.

## 1. Introduction

Lumbar degenerative disease (LDD) is the most common cause of mechanical low back pain, lower limb pain, and intermittent claudication [1]. When conservative treatment fails, lumbar interbody fusion is the standard surgical treatment for LDD [2, 3]. Although a satisfactory outcome can be expected with conventional open lumbar fusion surgery, extensive destruction of the posterior muscular-ligamentous complex can lead to significant postoperative pain, muscular atrophy, and functional disability in most patients [4, 5]. Compared to traditional open surgery, minimally invasive transforaminal lumbar interbody fusion (MIS-TLIF) uses tubular retractors for the surgical approach, which can significantly reduce tissue damage and preserve the physiological function of muscle tissue [6, 7]. Therefore, MIS-TLIF has been widely used for the treatment of LDD [8, 9]. However, there are still controversies about the internal fixation method and the implantation position of the cage for MIS-TLIF in clinical practice. Bilateral pedicle screw (BPS) fixation and oblique fusion cage placement are often used for lumbar fusion. However, the excessive rigidity of BPS fixation can cause device-related osteoporosis, absorption of grafted bone, and degeneration of adjacent segments [10, 11]. The oblique implantation of the cage into the intervertebral space may cause the risk of nerve compression due to the displacement and withdrawal of the cage along the original implantation way [12, 13]. Therefore, various improvement methods have been proposed. Of these, preliminary clinical evaluation has been performed for hybrid internal fixation (ipsilateral pedicle screw fixation and contralateral translaminar facet screw fixation) [14], as well as for horizontal placement of the fusion cage in the intervertebral space [15, 16]. However, there is still a lack of theoretical research on their combined application in MIS-TLIF.

The finite element analysis (FEA) can provide detailed data that is not influenced by complicated clinical factors, which is ideal to evaluate spinal biomechanics [17, 18]. Therefore, the FEA method was used in this study to compare the effects of hybrid internal fixation combined with cage horizontal placement fusion strategy and traditional fusion strategy on the biomechanics of the lumbar spine and to provide a theoretical basis for the application of this new fusion strategy in MIS-TLIF.

## 2. Materials and Methods

### 2.1. Lumbar Spine FE Model

Computed tomographic (CT) images of the lumbar spine, used as inputs for the development of the model, were obtained from a healthy 24-year-old male (70 kg, 176 cm, and no history of lumbar spine disease). Images were obtained using a Philips Brilliance 64 Slice CT scanner (Philips Medical Systems, Inc., OH, USA) at a slice interval of 0.625 mm. Images were saved in DICOM format and imported into Mimics research software (version 19.0; Materialise, Inc., Belgium) for preprocessing and to build a preliminary three-dimensional model of the L4-L5 lumbar segment. The file (format: .stl) generated by Mimics software was imported into Geomagic Wrap 2017 software (3D Systems, Inc., USA) for optimization and smoothing of the model. The file generated by the Geomagic software (.stp format) was imported into SolidWorks (version 2017, Dassault Systems, Inc., USA) to assemble the different components of the model: bones, annulus, nucleus pulposus, screws, and cages. The reconstructed model was saved (.X_T file). Finally, the. X_T file was imported into ANSYS software (version 20.0; ANSYS, Inc., USA) for finite element analysis.

**Figure 1:**
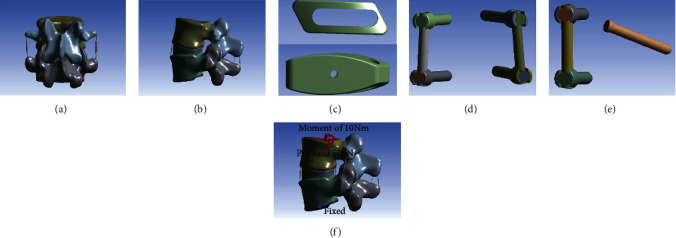
(a) Anterior-posterior and (b) lateral views of the L4-L5 reference model (Model-INT). (c) Model of the Z-Cage (size: 12 × 10 × 32 mm). (d) Model of the pedicle screws (size: 6.0 × 45 mm) and titanium rods (size: 5.5 × 40 mm). (e) Model of the translaminar facet screws (size: 4.5 × 50 mm). (f) A preload of 500 N and a moment of 10 Nm were applied to the models to simulate lumbar motion.

Due to the complex shape of the lumbar vertebrae model, the 3D tetrahedral elements were employed to mesh the FE model except for the ligaments. The vertebral body was divided into the outer cortical bone and inner cancellous bone. The thickness of cortical bone was 1.0 mm and the thickness of bone endplate was 0.5 mm [19], and the endplates were set on the superior and inferior surfaces of each vertebral body. The intervertebral disc was divided into nucleus pulposus and annulus fibrosus. According to the lumbar model, there was no separation between the annulus fibrosus and the nucleus pulposus under load, and no separation between the vertebral body and the disc under load; therefore, the interfacing of the nucleus pulposus and the annulus fibrosus and interfacing of the disc and the vertebral body were set as binding. The interfaces of vertebrae and cages were also assigned to tie constraints [19]. There were ligaments around the lumbar vertebral body, which can limit the range of motion of the vertebral body of the spine. However, because the model of the ligament is too slender and irregular in shape, a spring element is used in the model to simulate the ligament of the intervertebral body. The ligaments of the lumbar spine were included: the anterior longitudinal ligament (ALL), posterior longitudinal ligament (PLL), ligament flavum (LF), interspinous ligament (ISL), supraspinous ligament (SSL), intertransverse ligament (ITL), and joint capsule ligament (CL). The material properties were based on the previously reported values [20, 21]. Material properties used in the model (Model-INT), including Young's modulus, Poisson's ratio, and the cross-sectional areas of structures, are summarized in Table 1. The resultant reference model (Model-INT) is shown in Figures 1(a) and 1(b).

### 2.2. MIS-TLIF FE Model

The L4-L5 functional spinal unit was selected to evaluate the MIS-TLIF technique as it is the most frequent site of LDD requiring surgical treatment [22]. The steps of the MIS-TLIF procedure are as follows.

First, the left L4 lower articular process, part of the L5 upper articular process, the ligament flavum, and the posterolateral annulus fibrosus were removed. The nucleus pulposus tissues in the intervertebral disc could then be removed. A cage (size: 12 × 10 × 32 mm, Figure 1(c)) was fabricated based on the Z-Cage (WeGo Company, Shandong, China), using polyetheretherketone (*E* = 3.6 GPa). The pedicle screws (size: 6.0 × 45 mm, Figure 1(d)), translaminar facet screws (size: 4.5 × 50 mm, Figure 1(e)), and titanium rods (size: 5.5 × 40 mm) were fabricated based on the Premier Lumbar Internal Fixation System (WeGo Company, Shandong, China). All fixation components were made of titanium alloy (*E* = 110 Gpa).

**Figure 2:**
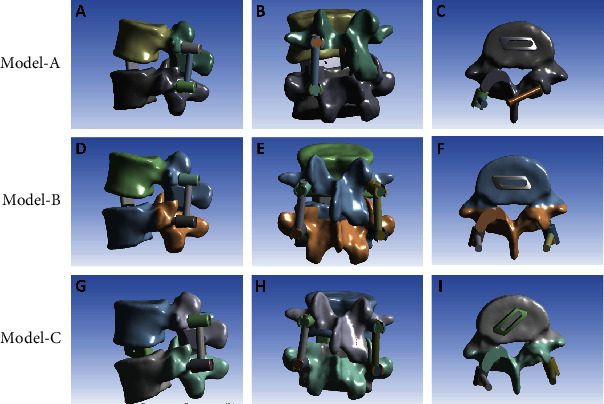
(a)–(c) Model-A, with hybrid internal fixation (ipsilateral pedicle screw fixation and contralateral translaminar facet screw fixation)+horizontal single cage implantation. (d)–(f) Model-B, with BPS fixation+horizontal single cage implantation. (g)–(i) Model-C, with BPS fixation+45° oblique single cage implantation.

All the MIS-TLIF FE models were constructed based on the validated Model-INT: Model-A, hybrid internal fixation+horizontal single cage implantation (Figures 2(a)–2(c)); Model-B, BPS fixation+horizontal single cage implantation (Figures 2(d)–2(f)); and Model-C, BPS fixation+oblique 45° single cage implantation (Figures 2(g)–2(i)). Unilateral pedicle screw fixation was not evaluated owing to a previous report showing poor biomechanical stability with this type of fixation [23].

Figures 2(a)–2(c) Model-A, with hybrid internal fixation (ipsilateral pedicle screw fixation and contralateral translaminar facet screw fixation)+horizontal single cage implantation. (d)–(f) Model-B, with BPS fixation+horizontal single cage implantation. (g)–(i) Model-C, with BPS fixation+45° oblique single cage implantation.

### 2.3. Loading and Boundary Conditions

All nodes of the L5 lower endplate and the two lower facet surfaces were set to be fully constrained with 0 degrees of freedom to ensure no displacement or rotation of L5 under external forces. A 500 N preload was applied to the upper endplate of L4 to simulate loading by the upper body weight (Figure 1(f)). A moment of 10 Nm was then applied to simulate the following physiological motions, as per previous studies [24, 25]: lumbar flexion (FL), extension (EX), left lateral bending (LLB), right lateral bending (RLB), left rotation (LR), and right rotation (RR). ROM is an important indicator of lumbar stability [26]. To compare the ROM between models, the ROM ratio was calculated using the Model-INT as the reference: ((Model − INT − Model − A/B/C) ÷ Model − INT) × 100%. The ROM and ROM ratio were calculated for each of the six directions of loading motions. The peak stress in the internal fixation system and cage was used as an index of the risk of fixation failure [27].

### 2.4. Statistical Analysis

Statistical analysis and graphing were performed using GraphPad Prism (version 7.0; GraphPad Software Inc., La Jolla, CA, USA). One-way analysis of variance (ANOVA) was used to evaluate differences in ROM, ROM ratio, and peak stress between the different internal fixation techniques and cage implantation position, with a *P* value <0.05 considered significant.

## 3. Results

### 3.1. Reliability of the Model-INT Model

**Figure 3:**
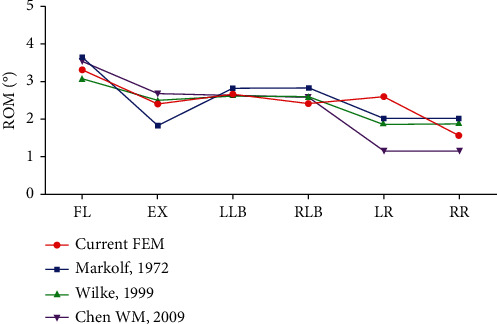
Range of motion Model-INT compared to previously reported values under the same conditions of loading. FL: Lumbar flexion; EX, Extension; LLB: Left lateral bending; RLB: Right lateral bending; LR: Left rotation; RR: Right rotation; ROM: Range of motion.

The reliability of the Model-INT was confirmed by ROM under preload conditions of a 500 N force and a moment of 10 Nm, which were comparable to values previously reported in experimental results [28–30] (Figure 3).

### 3.2. Range of Motion

**Figure 4:**
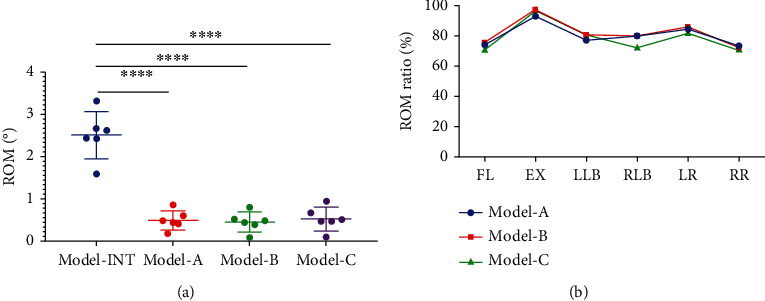
(a) The ROM values of Models A, B, and C were not different (*P* > 0.05) but they were significantly lower than the Model-INT for all loading motions (^∗∗∗∗^*P* < 0.0001). (b) The ROM ratio ranged between 71.07 and 97.53% for Models A, B, and C, but the model of the ROM ratio curves was highly similar. FL: Lumbar flexion; EX: Extension; LLB: Left lateral bending; RLB: Right lateral bending; LR: Left rotation; RR: Right rotation; ROM: Range of motion.

The ROM for all models (-INT, A, B, and C) under the six loading motions (FL, EX, LLB, RLB, LR, and RR) is shown in Figure 4(a). The reference values (Model-INT) were as follows: FL, 3.32°; EX, 2.43°; LLB, 2.66°; RLB, 2.42°; LR, 2.62°; and RR, 1.59°. The ROM for Models A, B, and C were not different (*P* > 0.05) but were significantly lower than the ROM of Model-INT (*P* < 0.0001 for all loading motions). The ROM ratio ranged between 71.07 and 97.53% for Models A, B, and C across all six loading motions (Figure 4(b)). Although there were subtle differences in the ROM ratio for Models A, B, and C, the trend in the ROM ratio was similar across all six loading motions. It can be found that the novel fusion strategy can achieve postoperative stability similar to the traditional fusion strategy.

### 3.3. Peak Stress in the Internal Fixation System

**Figure 5:**
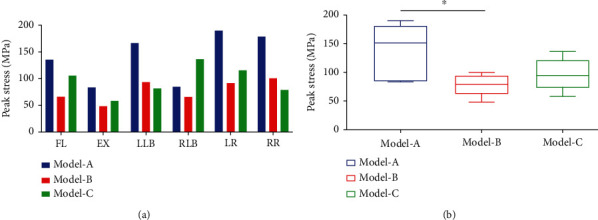
(a) The peak stress of the internal fixation system was higher in Model-A than in Models B and C for almost all loading motions, which was 83.26 MPa (EX) to 189.81 MPa (LR). (b) Although the average peak stress in Model-A was significantly higher than that of Models B and C, only the difference between Models A and B was significant (^∗^*P* < 0.05). FL: Lumbar flexion; EX: Extension; LLB: Left lateral bending; RLB: Right lateral bending; LR: Left rotation; RR: Right rotation.

The peak stress in the internal fixation system for all loading motions is shown in Figure 5(a). The range of peak stress was as follows: Model-A, 83.26 MPa (EX) to 189.81 MPa (LR); Model-B, 48.56 MPa (EX) to 100.09 MPa (RR); and Model-C, 58.10 MPa (EX) to 136.05 MPa (RLB). The peak stress was significantly higher in Model-A than in Models B and C. Specifically, the peak stress in Model-A was higher (fold-increase) than in Models B and C, respectively, in LLB (1.80- and 2.05-fold), LR (2.07- and 1.64-fold), and RR (1.79- and 2.28-fold). In addition, the peak stress in the internal fixation system was significantly lower in Model-B than in Models C and A in FL, EX, RLB, and LR. As shown in Figure 5(b), although the average peak stress in Model-A was significantly higher than that of Models B and C, only the difference between Models A and B was significant (*P* < 0.05). By comparing the values of Models B and C, it can be found that under the same internal fixation method, the horizontal placement of the cage reduces the peak stress of the internal fixation system.

### 3.4. Peak Stress in the Cage

**Figure 6:**
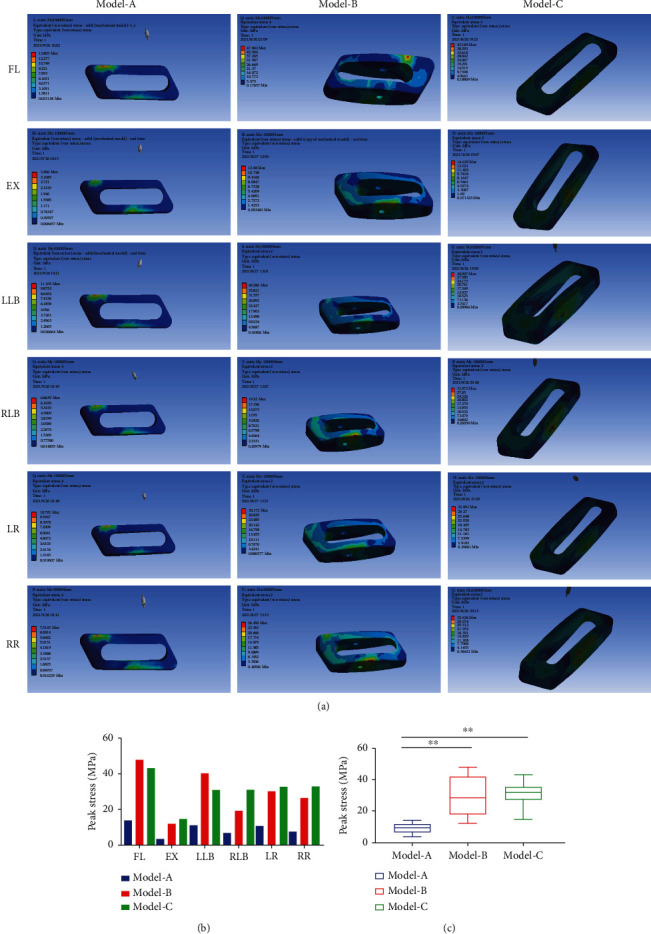
(a) The stress cloud diagrams showed that the peak stress of the implanted cage was concentrated in the area in contact with the vertebral endplate. (b) The peak stress in the implanted cage in Model-A was significantly lower than that of Models B and C for all loading motions. (c) The peak stress of the implanted cage in Model-A was significantly different from Models B and C (^∗∗^*P* < 0.01). FL: Lumbar flexion; EX: Extension; LLB: Left lateral bending; RLB: Right lateral bending; LR: Left rotation; RR: Right rotation.

The cloud diagram of the stress in implanted cages is shown in Figure 6(a). It can be found that the peak stress appears in the area where the cage and the endplate are in contact, which is in line with the actual clinical situation. The peak stress in the cage predicts the stress on the endplate due to the interaction of these forces. The peak stress in the cage is shown in Figure 6(b). The peak stress in Model-B reached maximum values in FL (47.86 MPa) and LLB (40.29 MPa). In Model-C, maximum peak stress was created in EX (14.64 MPa), RLB (31.07 MPa), LR (32.64 MPa), and RR (32.89 MPa). The peak stress in the cage in Model-A was obviously lower than that of Models B and C. Compared to Model-B, the peak cage stress in Model-A was 29% in FL and 28% in LLB. Compared to Model-C, the peak cage stress in Model-A was 24% in EX, 22% in RLB, 33% in LR, and 23% in RR. As shown in Figure 6(c), the peak cage stress in Model-A was significantly different from the peak cage stress in Models B and C for all loading motions (*P* < 0.01), with no difference between Models B and C (*P* > 0.05).

## 4. Discussion

Modern intervertebral fusion is mostly achieved by implanting pedicle screws and intervertebral cages, which play an important role in promoting intervertebral fusion and maintaining early biomechanical stability of treated segments [31]. In clinical practice, the fusion strategy mainly depends on the experience and preferences of the surgeon. However, it also causes many implant-related complications. The excessive rigidity of BPS fixation can cause device-related complications [10, 11]. At the same time, cage-related complications have become increasingly prominent, including cage displacement, subsidence, and nonfusion, with these complications yet to be effectively solved [12, 13]. The purpose of our study is important in this regard, providing biomechanical evidence to assist surgeons in selecting the appropriate fusion strategy for different conditions.

We evaluated the biomechanics for two MIS-TLIF internal fixation modes (hybrid internal and BPS) and two cage implantation methods (horizontal and oblique 45° implantation) using FEA. The ROM, ROM ratio, internal fixation system peak stress, and cage peak stress were estimated to identify the optimal internal fixation strategy for better biomechanical stability and a lower failure rate. Salient findings were as follows. First, with a horizontal cage placement, both hybrid (Model-A) and BPS (Model-B) internal fixation significantly reduced lumbar motions (Figure 4), achieving similar fixation strength, consistent with previous research conclusions [14, 32, 33]. However, the study found that the hybrid internal fixation bore greater peak stress than the BPS fixation (Figure 5), which may be closely related to the asymmetry in screw arrangement with the hybrid internal fixation. Since the predicted peak stress in the fixation components was much lower than the inherent yield strength of the titanium alloy material (877 ± 18.5 MPa) [34], the risk of failure of mixed internal fixation was not increased. Second, there was no significant difference in the fused segment stability for a horizontal (Model-B) and 45° oblique (Model-C) cage (Figure 4), but the horizontal cage position did reduce the peak stress in the internal fixation and (Figure 5). Thus, it could lower the risk of internal fixation failure. Previous clinical research has shown that horizontal placement of cages in lumbar fusion surgery can improve lumbar lordosis, restore spinal sagittal balance, and prevent fusion cage displacement [15, 16]. Theoretically, it is also less likely that a horizontally positioned cage would migrate from the intervertebral space than a cage placed at an oblique angle of 45°. It is extremely difficult that migration of the cage from its original position would allow rotation of the cage and exit from the intervertebral space. Third, the cloud diagram of stress distribution identified peak stress in the cage at the contact area between the cage and endplate. According to the principle of force interaction, it can be considered that the endplate is, therefore, subjected to the same magnitude of stress. This finding is consistent with clinical reality. The peak stress of the cage is significantly reduced in the hybrid internal fixation model (Model-A, Figure 6), which reflects the higher stress on the hybrid internal fixation than on BPS internal fixation (Model-B, Figure 5). The triangular structure of the hybrid internal fixation method provides excellent mechanical stability. Therefore, it can be inferred that the low peak stress of the cage reduces the stress shielding effect and reduces the risk of cage collapsing, which is especially suitable for application in older patients with osteoporosis.

The limitations of our study need to be acknowledged. First, the FE model of L4-L5 segments was constructed from CT images of a young male adult without evidence of spinal disease. Therefore, structural changes in the spine caused by LDD were not considered. Second, the FE model does not consider the influence of paravertebral muscles, which may have a slight influence on the stability of the lumbar spine.

## 5. Conclusion

According to the results of our FEA, hybrid internal fixation and horizontal single cage implantation can achieve the same biomechanical stability as the traditional fixation method by open surgery while significantly reducing the peak stress in the cage and vertebral endplate. At the same time, the approach can reduce surgical damage as much as feasible, which is in line with the concept of minimally invasive surgery. Based on our results, we propose that the hybrid internal fixation and horizontal single cage implantation strategy is expected to become an ideal choice for MIS-TLIF.

## Figures and Tables

**Table 1:** Summary of material properties used in finite element models.

Material properties	Young's modulus (MPa)	Poisson's ratio	Cross section area (mm^2^)
Cortical bone	12000	0.3	—
Cancellous bone	100	0.2	—
Endplate	4000	0.3	—
Posterior bone	3500	0.25	—
Articular cartilage	25	0.25	—
Annulus fibrosus	6	0.40	—
Nucleus pulposus	1	0.50	—
ALL	7.8	—	22.4
PLL	1	—	7.0
LF	1.7	—	14.1
ITL	1	—	0.6
CL	7.5	—	10.5
ISL	1	—	14.1
SSL	8	—	10.5
Cage (PEEK material)	3500	0.3	
Screws and rods (titanium alloy material)	110000	0.3	

ALL: Anterior longitudinal ligament; PLL: Posterior longitudinal ligament; LF: Ligament flavum; ISL: Interspinous ligament; SSL: Supraspinous ligament; ITL: Intertransverse ligament; CL: Joint capsule ligament.

## Data Availability

The data used to support the findings of this study are included within the article.
